# Hyperparameter optimization to enhance the performance of deep learning models for the early detection of invasive turtles in Korea

**DOI:** 10.1038/s41598-026-37636-2

**Published:** 2026-02-06

**Authors:** Jong-Won Baek, Jung-Il Kim, Min-Ho Mun, Chang-Bae Kim

**Affiliations:** 1 Biotechnology Major, Sangmyung University, Seoul, 03016 Korea; 2 Ocean Climate Response and Ecosystem Research Department, Korea Institute of Ocean Science and Technology, Busan, 49111 Korea

**Keywords:** Invasive turtle, Early detection, Deep learning, Object detection, Hyperparameter optimization, Computational biology and bioinformatics, Ecology, Ecology, Engineering, Mathematics and computing

## Abstract

Invasive freshwater turtles are major drivers of biodiversity loss, underscoring the importance of early detection and management. However, it is challenging for experts to manually monitor a broad geographic area, necessitating support tools. Deep learning-based object detection models have displayed high performance in automating wildlife monitoring tasks. Furthermore, hyperparameter optimization, including optimizer selection and hyperparameter tuning, might further enhance performance by optimizing training settings to the dataset. In this study, an optimized model was developed to apply hyperparameter optimization to detect and classify six invasive turtle species in Korea from images. The optimized model was compared to a default model trained using the default optimizer and hyperparameters. The optimized model outperformed the default model, as indicated by the evaluations of mean average precision using a fixed intersection over union threshold of 0.5 (0.973 vs. 0.959) and a range of thresholds ranging from 0.5 to 0.95 (0.841 vs. 0.815). The classification accuracy of the optimized model reached 92.7%, exceeding that of the default model (89.9%). These findings highlight the utility of hyperparameter optimization and suggest that the proposed approach can support the early detection of invasive turtles, thereby enhancing to invasive species management.

**Supplementary Information:**

The online version contains supplementary material available at 10.1038/s41598-026-37636-2.

## Introduction

Invasive species are major drivers of biodiversity decline globally^1^. The introduction and subsequent spread of these species are facilitated primarily by the global wildlife trade, which facilitates these movement across ecosystems^2^. These species adversely affect native species through competition, ecosystem alteration, and pathogen spread, hindering biodiversity conservation^3–6^. Moreover, these species rapidly adapt to new environments and they can spread widely^7^. Among these, invasive freshwater turtles could have particularly profound effects because of these long lifespans and adaptability to suboptimal environments^8,9^. Moreover, climate change is further exacerbating this issue. As ambient and water temperatures increase, heat-tolerant freshwater turtles from temperate and tropical regions are becoming more likely to outcompete native species, increasing the likelihood that invasive turtles will successfully establish themselves in new habitats^10^. For instance, *Trachemys scripta*, a globally invasive freshwater turtle with relatively high thermal tolerance, may gain a competitive advantage over native Chinese species such as *Pelodiscus sinensis* and *Mauremys reevesii* under warmer conditions^10^. Indeed, several Asian countries have reported the establishment of invasive freshwater turtles or the potential for such establishment, highlighting the necessity for effective management^11,12^. *Trachemys scripta* and *Chelydra serpentina* have been reported as established invasive species or identified as having high invasive potential in China, Japan, and Taiwan^11–14^. Additionally, *Macrochelys temminckii* has been detected as an invasive species in China and Taiwan, and *Pseudemys concinna* has been noted as an invasive species in Taiwan^11,13^. Along with these four species, *Mauremys sinensis* and *P. nelsoni* have been managed as invasive species in Korea^15^. Therefore, management is necessary to prevent further establishment of invasive turtles in domestic ecosystems and nationwide spread.

Effective management of invasive species critically relies on early detection through monitoring^16,17^. Such early detection might minimize ecological and economic damage^17^. Typically, early detection of invasive species is achieved through proactive field monitoring^18^. However, conventional invasive species detection depend heavily on field surveys conducted by experts^19,20^. Although this method is highly accurate, it is labor-intensive and limited in geographic coverage^21^. Recent studies explored broader surveillance methods using unmanned tools such as drones and camera traps^22,23^. Nevertheless, manual validation of vast amounts of submitted data remains a bottleneck because of the time-consuming nature of the analysis and requirement for accurate species classification^17,24^. Therefore, the demand for deep learning approaches to support rapid and accurate species detection and classification by morphological experts is increasing^24^. Representatively, convolutional neural network-based object detection models have emerged as automated tools for identification using images captured by drones and camera traps^25–27^. These models can detect and classify objects simultaneously within image datasets^28^. Furthermore, these models have been considered efficient because of these balances of speed, accuracy, and versatility for ecological monitoring, including species detection and classification tasks^22,29–31^. These developments offer the potential to streamline data processing and reduce the labor required for manual review^24,32^.

Supporting manual validation and streamlining invasive species monitoring using object detection models fundamentally requires high model performance, including object detection and classification. However, the available models generally use default hyperparameters that are set to universal values for benchmark datasets, which consists primarily of general objects such as trains, cups, and books^29^. The default hyperparameters of this dataset might be unsuitable for datasets focused on biological objects. Therefore, to achieve high model performance, it is necessary to use appropriate hyperparameter values for the biological dataset. Hyperparameter optimization is the process of identifying the most effective combination of hyperparameters, which control the learning process, to optimize model performance^33^. This optimization enhances the detection of small objects and improves classification accuracy, thereby increasing overall model performance^34,35^. For this reason, several studies have applied hyperparameter optimization to improve model performance.

In studies focusing on hyperparameter optimization for biological detection and classification, researchers both compared various optimizers to identify the most effective ones and employed hyperparameter tuning across multiple hyperparameter combinations to enhance model performance. For instance, Isa et al. (2021) applied hyperparameter optimization to improve underwater animal detection using You Only Look Once (YOLO) v5 model. Specifically, the researchers compared the default setting—stochastic gradient descent (SGD) optimizer with a learning rate 0.01 and momentum 0.937— with optimized setting using the adaptive moment estimation (Adam) optimizer with a manually adjusted learning rate of 0.0001 and momentum of 0.9^36^. Their study demonstrated improvements in detection and classification performance, with mean average precision (mAP) increasing from the baseline of 0.977 (default settings) to a peak of 0.986 following hyperparameter optimization. However, manual adjustment of hyperparameters was challenging in this study, primarily because of the difficulty in systematically identifying optimal ranges and values. To enhance the detection performance of wild animals such as wild boars and deer using thermal images, Popek et al. (2023) employed YOLOv5 model and applied evolutionary algorithms, to adjust two hyperparameters from default setting (learning rate 0.01 and momentum 0.937) to a learning rate of 0.0123 momentum of 0.934^37^. The performance evaluation indicated that this hyperparameter adjustment did not yield a meaningful improvement over the default setting. This study was limited to exploring a small set of hyperparameters using evolutionary algorithms. Although these automated algorithms increase the probability of identifying optimal hyperparameter values, they become progressively time-consuming and resource-intensive when large numbers of hyperparameters are involved. However, random search can avoid exhaustive searches in less promising regions, thus significantly reducing computational costs and time^38^. This might be particularly advantageous for optimizing a large number of hyperparameter. Therefore, to efficiently identify optimal hyperparameter combinations across a broader range and large number of parameters, methods such as random search, which strategically sample the search space, should be considered.

This study aims were: (1) to identify the most suitable optimizer for the invasive turtle dataset among the optimizers provided, (2) to determine the optimal combination of hyperparameters through hyperparameter tuning using a random search approach in the best-performing optimized model, and (3) to compare the performance of the model trained with the default optimizer and hyperparameters to that of the model optimized through both optimizer selection and hyperparameter tuning.

## Materials and methods

**Fig. 1:**
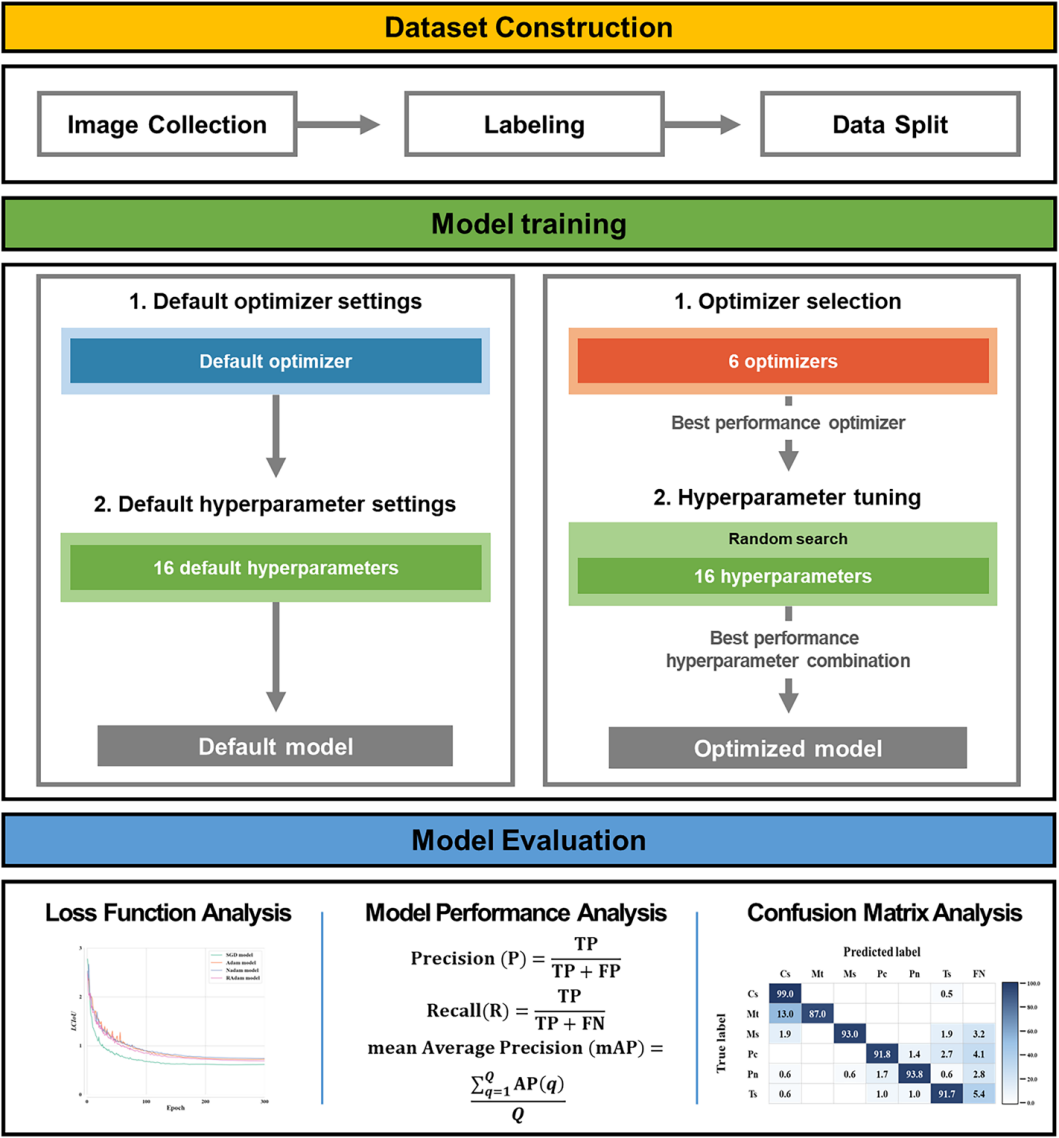
Overview of the workflow in this study.

Figure 1 presents a flowchart of the entire methodology of this study. Images of the six invasive turtles were collected and labeled to create a final dataset for model training. Six optimizers were compared for model training, and the best-performing optimizer was selected. Then, 16 hyperparameters were optimized via random search. Model training was conducted using training and validation sets in this study, and model performance was evaluated using the test set.

### Data collection

As no standardized dataset exists for invasive turtle species, images of six invasive turtle species found in Korea (*C. serpentina*, *M. temminckii*, *M. sinensis*, *P. concinna*, *P. nelsoni*, and *T. scripta*) were collected from iNaturalist (https://www.inaturalist.org), which provides images taken by citizen scientists. Images were obtained using the inat_images R package, and only research-grade images were included in the setting value to ensure data quality^39^. Although the images of iNaturalist had been verified by curators, those included dead individuals, out of focus, and misidentified images. Therefore, all images were re-verified by the authors to establish a clearer dataset. In particular, images suspected of being misidentified were verified using species-specific morphological characteristics described in the taxonomic literature^40–42^. During this process, freshwater turtle images were first screened based on morphological characteristics of the carapace and plastron, which are widely regarded as primary morphological traits for species identification, and were subsequently verified using morphological characteristics of the head and neck in a stepwise manner. Images that could not be reliably identified using these features were excluded. All images used in this study had minimum dimensions of 500 × 500 pixels with a resolution of 72 dpi. The dataset, which was curated to ensure robustness across diverse environmental conditions, included images taken from various angles, with diverse backgrounds, multiple individuals, and multiple species, to train robustness models. The number of images per species is summarized in Table 1. The entire turtle body was designated as the region of interest, since key morphological features, such as the carapace, plastron, and snout, are present throughout the body. Bounding boxes were manually annotated using LabelImg^43^. The final dataset was randomly split into training (4,437 images [64%]), validation (1,106 images [16%]), and test sets (1,395 images [20%]; Table 1).

**Table 1:** Summary of the dataset comprising six invasive turtle species.

Species	Training set	Validation set	Test set
*Chelydra serpentina*	1,543	385	483
*Macrochelys temminckii*	184	46	59
*Mauremys sinensis*	474	118	149
*Pseudemys concinna*	539	134	170
*Pseudemys nelsoni*	643	160	203
*Trachemys scripta*	1,054	263	331
Total	4,437	1,106	1,395

### Model training

The YOLO11 model exists in five versions based on network depth and width: n, s, m, l, and x. Among them, YOLO11s has displayed superior accuracy while maintaining fast inference speed, prompting its use in this study^44^. The experimental environment for hyperparameter optimization, including optimizer selection and hyperparameter tuning, consisted of a Rocky Linux 8 operating system, two Intel Xeon Gold 6326 CPUs, eight 64 GB REG.ECC DDR4 SDRAM modules, and an NVIDIA RTX A5000 GPU with 24 GB of memory. The training environment was configured using Python 3.8.2, CUDA 12.2, cuDNN 8.9.3, and PyTorch 2.0.1. To prevent overfitting during training, data augmentation was performed using Albumentations^45^ and Mosaic Augmentation^46^. Albumentations involve adjustments to the images’ hue, saturation, and brightness of images, as well as translation, scaling and flipping^45^. Mosaic Augmentation is a method of combining four images into a single image^46^.

### Optimizer selection

For optimizer selection, the YOLO11 model trained with six different optimizers was evaluated in this study: SGD, Adam, Adam with decoupled weight decay (AdamW), Nesterov-accelerated Adam (Nadam), rectified Adam (RAdam), and root mean square propagation (RMSProp). A brief summary of the six optimizers is provided in Table S1. During this process, the YOLO11s model was trained from scratch, without COCO-pretrained weights, separately using each of the six optimizers and compared using a 640 × 640 input image size, batch size of 16, and 300 training epochs. The original images were automatically resized to 640 × 640 size by the YOLO data loader. This size is the standard YOLO configuration, and is independent of any pretrained weights. After comparing the six optimizers, the optimizer with the best performance was selected based on the mAP value, and the model was subsequently optimized.

### Hyperparameter tuning

For hyperparameter tuning, a random search algorithm was employed to identify the optimal hyperparameter settings. The input size (640 × 640), batch size (16), and number of epochs (300) were unchanged from optimizer selection, with 300 iterations conducted for the random search. The list of 16 hyperparameters used for hyperparameter tuning is presented in Table 2. In this random search, one iteration corresponded to randomly sampling a single configuration of the 16 tuned hyperparameters from predefined ranges, training the YOLO11s model with that configuration for 300 epochs. The predefined ranges of each hyperparameter are presented in Table S2. A total of 300 such configurations were evaluated and the hyperparameter configuration which showed the highest mAP value were used to train the optimized model. The default model was trained using the default hyperparameter settings with the same input size, batch size, and number of epochs as the optimized model.

**Table 2:** Hyperparameter configurations applied during hyperparameter tuning.

Hyperparameter	Description
lr0	Initial learning rate
lrf	Final learning rate
momentum	Momentum factor for SGD or beta1 for Adam optimizers
weight_decay	L2 regularization term
warmup_epochs	Number of epochs for learning rate warmup
warmup_momentum	Initial momentum for warmup phase
box	Weight of the Complete Intersection over Union loss function
cls	Weight of the classification loss function
dfl	Weight of the distribution focal loss function
hsv_h	Adjusting the hue of the image by a color wheel to introduce color variability
hsv_s	Altering the saturation of the image
hsv_v	Modifying the value (brightness) of the image
translate	Translating the image horizontally and vertically
scale	Scaling the image by a gain factor
fliplr	Flipping the image left to right with the specified probability
mosaic	Combining four training images into a single image

## Model evaluation

### Evaluation of model performance during training process

During the training process, model performance was monitored using three loss functions, in the training and validation sets. These loss functions quantify the discrepancy between predicted and actual outcomes, providing critical feedback that allows the model to iteratively refine its parameters and improve performance^29^. Complete intersection over union (*CIoU*) loss (*LCIoU*) measures the dissimilarity between predicted bounding boxes and the ground truth. It is computed according to *CIoU*, which incorporates the overlap area, the distance between the centers of the boxes, and the aspect ratio. This loss penalizes both localization inaccuracy and geometric inconsistency, leading to more precise and stable object localization^47^. Classification loss (*Lcls*) represents the average loss associated with classifying detected objects. It ensures that each object is accurately assigned to one of the predefined classes. Distribution focal loss (*Ldfl*) is applied to bounding box regression. Unlike traditional methods that predict bounding box coordinates directly, *Ldfl* predicts a probability distribution over discrete box offset. This approach enables the model to capture uncertainty in localization, thereby improving regression accuracy. Although both *Lcls* and *Ldfl* are based on cross-entropy formulations, their roles differ. *Lcls* performs standard multiclass classification for object categories^29^, whereas *Ldfl* uses weighted cross-entropy loss to regress continuous bounding box coordinates via discrete distribution modeling^48^. The computation of *LCIoU* was derived from the intersection over union (IoU) metric, as defined in Eq. (1), where *G* and *P* denote the ground-truth and predicted bounding boxes, respectively. The term $\:\nu\:$ describes the degree of aspect ratio consistency between the two boxes and is defined in Eq. (2), where $\:{w}^{gt}$ and $\:{h}^{gt}$ indicate the width and height of the ground-truth bounding box, and $\:w$ and $\:h$ denote the width and height of the predicted bounding box, respectively. The parameter $\:\alpha\:$ is a balancing factor determined using Eq. (3). *LCIoU* was then calculated using Eq. (4), in which $\:d$ refers to the distance between the center points of the two boxes, and $\:c$ represents the diagonal length of the smallest enclosing box that contains both boxes.

1 $$\:IoU=\frac{G\cap\:P}{G\cup\:P}$$

2 $$\:v=\frac{4}{{\pi\:}^{2}}{(arctan\frac{{w}^{gt}}{{h}^{gt}}-arctan\frac{w}{h})}^{2}$$

3 $$\:\alpha\:=\frac{v}{\left(1-IoU\right)+v}$$

4 $$\:{L}_{CIoU}=1-IoU+\frac{{d}^{2}}{{c}^{2}}+\alpha\:v$$

### Evaluation of model performance after training completion

After the training process, precision, recall, and mAP were used to evaluate model performance in the test set^29^. Precision denotes the fraction of positive predictions that are accurately identified by the model (5), whereas recall indicates the fraction of actual positives that the model correctly detects (6). Average precision (AP) was computed using Eq. (7), with n indicating the total number of ground truth objects. AP balances both precision and recall by measuring the area under the precision–recall curve, thereby supporting both object detection and classification evaluation. mAP was subsequently calculated using Eq. (8), where *Q* represents the total number of queries in the dataset, and AP(*q*) is to the AP score for each query *q*. In this study, two versions of mAP were assessed: mAP0.5 and mAP0.5–0.95. The former uses a fixed IoU threshold of 0.5, whereas the latter computes the value across multiple IoU thresholds ranging from 0.5 to 0.95. IoU was utilized to determine true positives by comparing the overlap ratio between manually annotated ground truth bounding boxes and those predicted by the model. Inference time was measured as the duration required to process one image. Additionally, a confusion matrix was constructed to summarize the model’s classification performance.

5 $$\:\mathrm{P}\mathrm{r}\mathrm{e}\mathrm{c}\mathrm{i}\mathrm{s}\mathrm{i}\mathrm{o}\mathrm{n}=\frac{\mathrm{T}\mathrm{r}\mathrm{u}\mathrm{e}\:\mathrm{P}\mathrm{o}\mathrm{s}\mathrm{i}\mathrm{t}\mathrm{i}\mathrm{v}\mathrm{e}}{\mathrm{T}\mathrm{r}\mathrm{u}\mathrm{e}\:\mathrm{P}\mathrm{o}\mathrm{s}\mathrm{i}\mathrm{t}\mathrm{i}\mathrm{v}\mathrm{e}+\mathrm{F}\mathrm{a}\mathrm{l}\mathrm{s}\mathrm{e}\:\mathrm{P}\mathrm{o}\mathrm{s}\mathrm{i}\mathrm{t}\mathrm{i}\mathrm{v}\mathrm{e}}$$

6 $$\:\mathrm{R}\mathrm{e}\mathrm{c}\mathrm{a}\mathrm{l}\mathrm{l}=\frac{\mathrm{T}\mathrm{r}\mathrm{u}\mathrm{e}\:\mathrm{P}\mathrm{o}\mathrm{s}\mathrm{i}\mathrm{t}\mathrm{i}\mathrm{v}\mathrm{e}}{\mathrm{T}\mathrm{r}\mathrm{u}\mathrm{e}\:\mathrm{P}\mathrm{o}\mathrm{s}\mathrm{i}\mathrm{t}\mathrm{i}\mathrm{v}\mathrm{e}+\mathrm{F}\mathrm{a}\mathrm{l}\mathrm{s}\mathrm{e}\:\mathrm{N}\mathrm{e}\mathrm{g}\mathrm{a}\mathrm{t}\mathrm{i}\mathrm{v}\mathrm{e}}$$

7 $$\:\mathrm{A}\mathrm{v}\mathrm{e}\mathrm{r}\mathrm{a}\mathrm{g}\mathrm{e}\:\mathrm{P}\mathrm{r}\mathrm{e}\mathrm{c}\mathrm{i}\mathrm{s}\mathrm{i}\mathrm{o}\mathrm{n}\:\left(\mathrm{A}\mathrm{P}\right)=\:\sum\:_{x=0}^{x=n-1}\{\mathrm{R}\mathrm{e}\mathrm{c}\mathrm{a}\mathrm{l}\mathrm{l}\left(x\right)-\mathrm{R}\mathrm{e}\mathrm{c}\mathrm{a}\mathrm{l}\mathrm{l}\left(x+1\right)\}\times\:\mathrm{P}\mathrm{r}\mathrm{e}\mathrm{c}\mathrm{i}\mathrm{s}\mathrm{i}\mathrm{o}\mathrm{n}\left(x\right)$$

8 $$\:\mathrm{m}\mathrm{e}\mathrm{a}\mathrm{n}\:\mathrm{A}\mathrm{v}\mathrm{e}\mathrm{r}\mathrm{a}\mathrm{g}\mathrm{e}\:\mathrm{P}\mathrm{r}\mathrm{e}\mathrm{c}\mathrm{i}\mathrm{s}\mathrm{i}\mathrm{o}\mathrm{n}\:\left(\mathrm{m}\mathrm{A}\mathrm{P}\right)=\frac{\sum\:_{q=1}^{Q}\mathrm{A}\mathrm{P}\left(q\right)}{Q}$$

## Results

### Optimizer selection and hyperparameter tuning

**Fig. 2:**
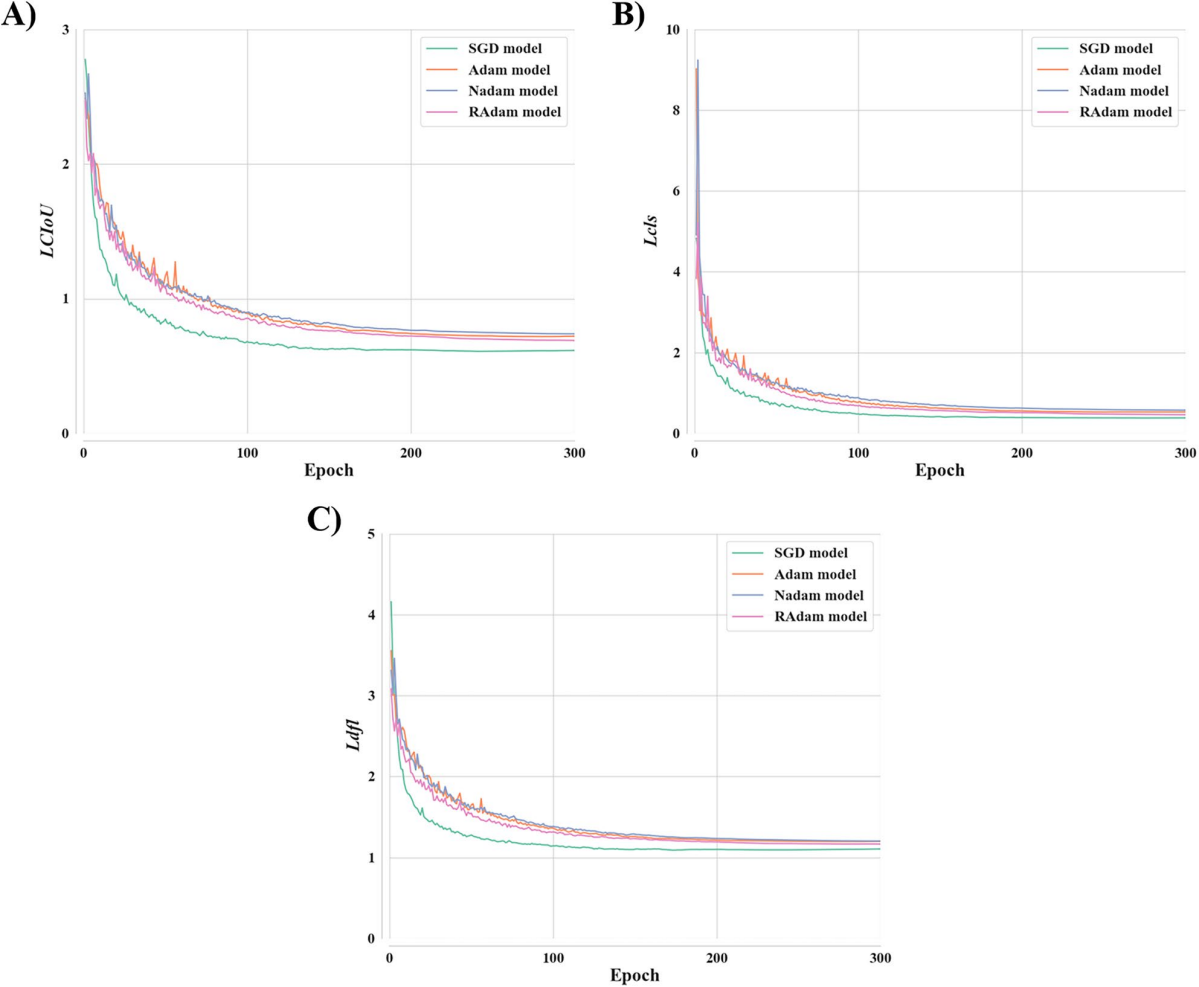
Comparison of validation loss function results for models trained with different optimizers. AdamW was excluded due to signs of overfitting, and RMSProp was excluded because of numerical instability in the loss function. (**A**) Complete Intersection over Union loss, *LCIoU*, (**B**) Classification loss, *Lcls*, (**C**) Distribution focal loss, *Ldfl*.

Six optimizers were applied to the YOLO11s model during optimizer selection, and the performance of the resulting models (SGD, Adam, AdamW, Nadam, RAdam, and RMSProp models) was compared. For the AdamW model, all three validation loss functions initially decreased during the early epochs but began to diverge and increase continuously after around the 55th epoch, while the training loss functions continued to decrease (Figure S1). This prolonged upward trend in losses during validation, when considered in conjunction with the decreasing trend in losses during training, indicates that overfitting occurred. The RMSProp model failed to reduce any of the loss functions throughout training; instead, the values increased, eventually reach a NaN value (Figure S2). This outcome typically indicates numerical instability in the loss function and an ineffective learning process^29^. Therefore, these two models were excluded from further analysis in this study. The loss function results indicated that *LCIoU* was lowest and most stable for the SGD model. Conversely, the value was relatively stable for the Nadam model, albeit with a consistently higher value than those of the other models (Fig. 2A). For *Lcls*, the SGD model converged the fastest and achieved the lowest value (Fig. 2B). Although *Lcls* gradually declined for the Nadam model, this model ultimately recorded the highest value among the examined models. Regarding *Ldfl*, the SGD model again outperformed the other models by maintaining both lower and more stable values. Conversely, the Nadam model exhibited the highest *Ldfl* (Fig. 2C). Overall, the SGD model exhibited the lowest values across all three loss functions and maintained stable convergence behavior throughout training than the models trained with the other optimizers.

**Fig. 3:**
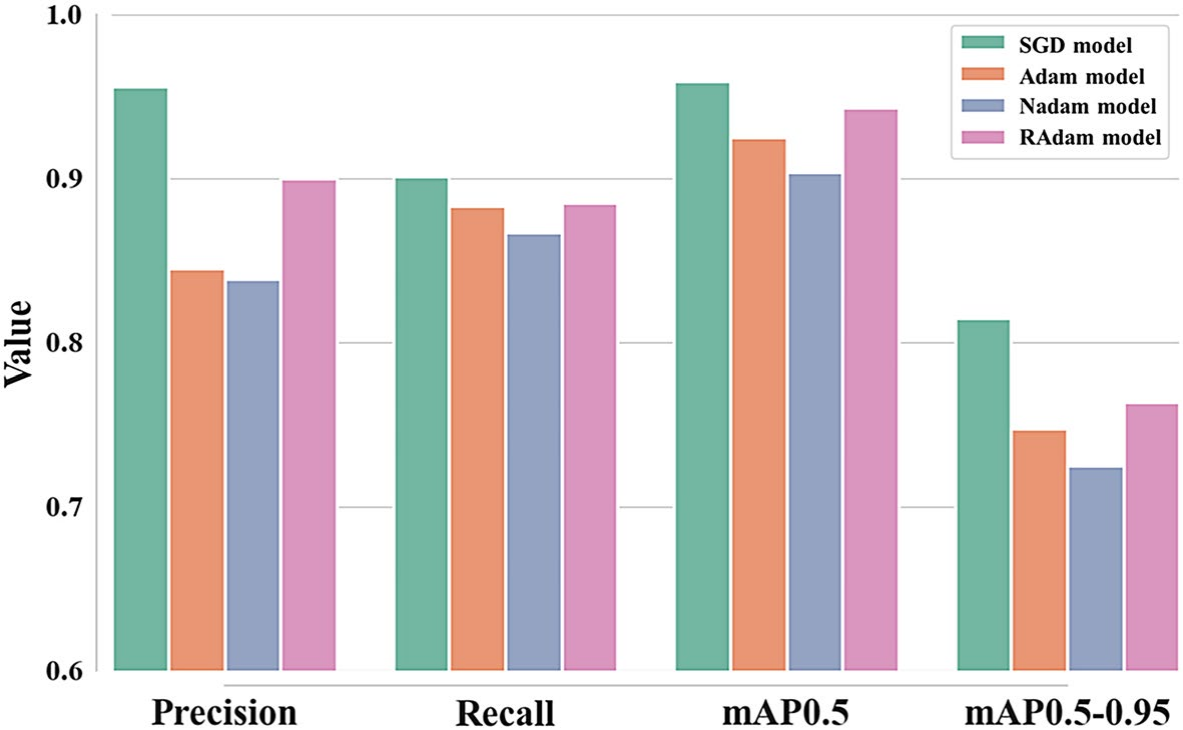
Comparison of precision, recall, mAP0.5, and mAP0.5–0.95, for models trained with different optimizers. AdamW model and RMSProp model were excluded due to low reliability in the evaluation of the loss function during training.

Throughout training, the loss functions for all four models decreased smoothly and consistently over the epochs for *LCIoU*, *Lcls*, and *Ldfl*. None of these models displayed signs of divergence or continual increase. The precision, recall, mAP0.5, and mAP0.5–0.95 of the four models are presented in Fig. 3. Precision was lowest for the Nadam model (0.839) and highest for the SGD model (0.956). Similarly, recall was lowest for the Nadam model (0.867) and highest for the SGD model (0.901). mAP0.5 was highest (0.959) for the SGD model and lowest (0.904) for the Nadam model. Furthermore, mAP0.5–0.95 was highest for the SGD model (0.815) and lowest for the Nadam model (0.725). The inference times of the models ranged from 0.0033 s for SGD to 0.0037 s for Adam and RAdam. Overall, these results indicate that the SGD optimizer was most suitable for analyzing the dataset of six invasive turtles of this study.

Hyperparameter tuning was conducted using the SGD model which selected as best optimizer based on mAP value. The optimal hyperparameter values were identified at the 114th iteration, yielding the highest performance among 300 iterations (Figure S3). The hyperparameter values at each iteration are presented in Figure S4. The default hyperparameter values based on the COCO dataset and the optimized hyperparameter values obtained through random search are presented in Table 3. After hyperparameter tuning, all 16 hyperparameters were adjusted, with five parameters (cls, weight_decay, mosaic, hsv_v, and initial learning rate [lr0]) exhibiting particularly notable shifts. cls increased from 0.50000 (default) to 0.77211 (optimized), representing the largest change (54.4%). weight_decay rose from 0.00050 to 0.00076 (52.0%). Conversely, mosaic decreased by 46.7% from 1.00000 to 0.53319, whereas hsv_v declined by 32.1% from 0.40000 to 0.27159. lr0 also declined by 31.2% from 0.01000 to 0.00688.

**Table 3:** A comparison of the hyperparameter values for the default and optimized models.

Hyperparameter	Default model	Optimized model
lr0	0.01000	0.00688
lrf	0.01000	0.01122
momentum	0.93700	0.96821
weight_decay	0.00050	0.00076
warmup_epoch	3.00000	3.45669
warmup_momentum	0.80000	0.93534
box	7.50000	6.28481
cls	0.50000	0.77211
dfl	1.50000	1.33119
hsv_h	0.01500	0.01087
hsv_s	0.70000	0.82473
hsv_v	0.40000	0.27159
translate	0.10000	0.12203
scale	0.50000	0.57497
fliplr	0.50000	0.39212
mosaic	1.00000	0.53319

### Comparative results default and optimized models

**Fig. 4:**
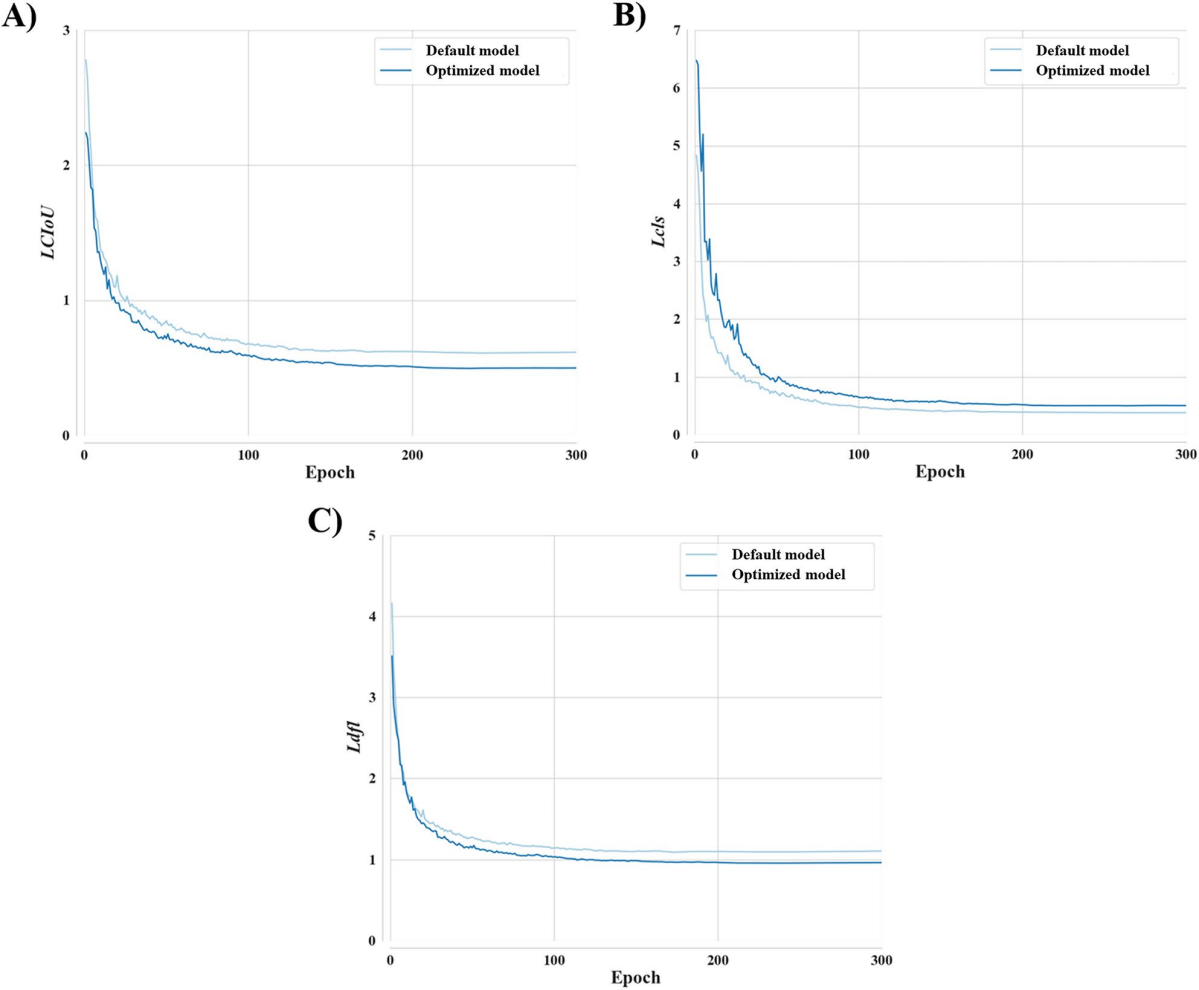
Loss function values for the default and optimized models. **A**) Complete Intersection over Union loss, *LCIoU*, **B**) Classification loss, *Lcls*, **C**) Distribution focal loss, *Ldfl*.

The optimized model was optimized using the SGD optimizer, which demonstrated the highest performance, whereas the default model was trained with the default optimizer (SGD optimizer). The default model used 16 default hyperparameter values, whereas the optimized model used 16 adjusted hyperparameter values. The training times of the default and optimized models were 5.072 and 5.080 h, respectively. The training losses across epochs for both models are presented in Fig. 4. Table S3 summarizes the loss function values at the epoch of the best performance, namely epoch 258 for the default model and epoch 295 for the optimized model. For both models, all three loss functions decreased steadily and converged without signs of overfitting. *LCIoU* at the best epoch was 0.61014 for the default model, versus 0.49867 for the optimized model (Fig. 4A). *Lcls* at the best epoch was 0.37940 for the default model, compared with 0.50189 for the optimized model (Fig. 4B), indicating better performance for the default model concerning classification loss. *Ldfl* at the best epoch was 1.09574 for the default model, versus 0.96152 for the optimized model. However, *Ldfl* exhibited faster and more consistent convergence to a lower value across epochs for the optimized model than for the default model (Fig. 4C).

Precision, recall, mAP0.5, and mAP0.5–0.95 for the two models are presented in Table 4. The overall precision values were 0.956 and 0.949 for the default and optimized models, respectively. For the default model, precision ranged from 0.942 for *T. scripta* to 0.975 for *M. temminckii*. In the optimized model, precision ranged from 0.913 for *T. scripta* to 0.976 for both *M. temminckii* and *P. nelsoni*. Recall was 0.901 for the default model, versus 0.934 for the optimized model. Recall was lowest for *M. temminckii* in both the default (0.853) and optimized models (0.889). Conversely, recall was highest for *C. serpentina* in the default and optimized models (0.984 and 0.990, respectively). mAP0.5 was 0.973 for the optimized model, outperforming the default model (0.959). In the optimized model, AP0.5 ranged from 0.959 for *T. scripta* to 0.993 for *C. serpentina*. The values for the default model ranged from 0.924 for *T. scripta* to 0.993 for *C. serpentina*. mAP0.5–0.95 was 0.841 the optimized model, compared with 0.815 for the default model. In the optimized model, AP0.5-0.95 ranged from 0.795 for *M. sinensis* to 0.900 for *C. serpentina*, whereas in the default model, the values for the same species were 0.788 and 0.881, respectively. The inference times were 0.0033 and 0.0041 s for the default and optimized models, respectively.

**Table 4:** Comparison of the performance of the default and optimized models.

Species	Precision		Recall		mAP0.5		mAP0.5–0.95	
	Default model	Optimized model	Default model	Optimized model	Default model	Optimized model	Default model	Optimized model
*Chelydra serpentina*	0.962	0.962	0.984	0.990	0.993	0.993	0.881	0.900
*Macrochelys temminckii*	0.975	0.976	0.853	0.889	0.960	0.975	0.793	0.816
*Mauremys sinensis*	0.949	0.942	0.917	0.935	0.960	0.963	0.788	0.795
*Pseudemys concinna*	0.955	0.925	0.870	0.932	0.951	0.969	0.835	0.866
*Pseudemys nelsoni*	0.952	0.976	0.927	0.938	0.962	0.978	0.801	0.831
*Trachemys scripta*	0.942	0.913	0.856	0.923	0.924	0.959	0.789	0.836
Total	0.956	0.949	0.901	0.934	0.959	0.973	0.815	0.841

**Fig. 5:**
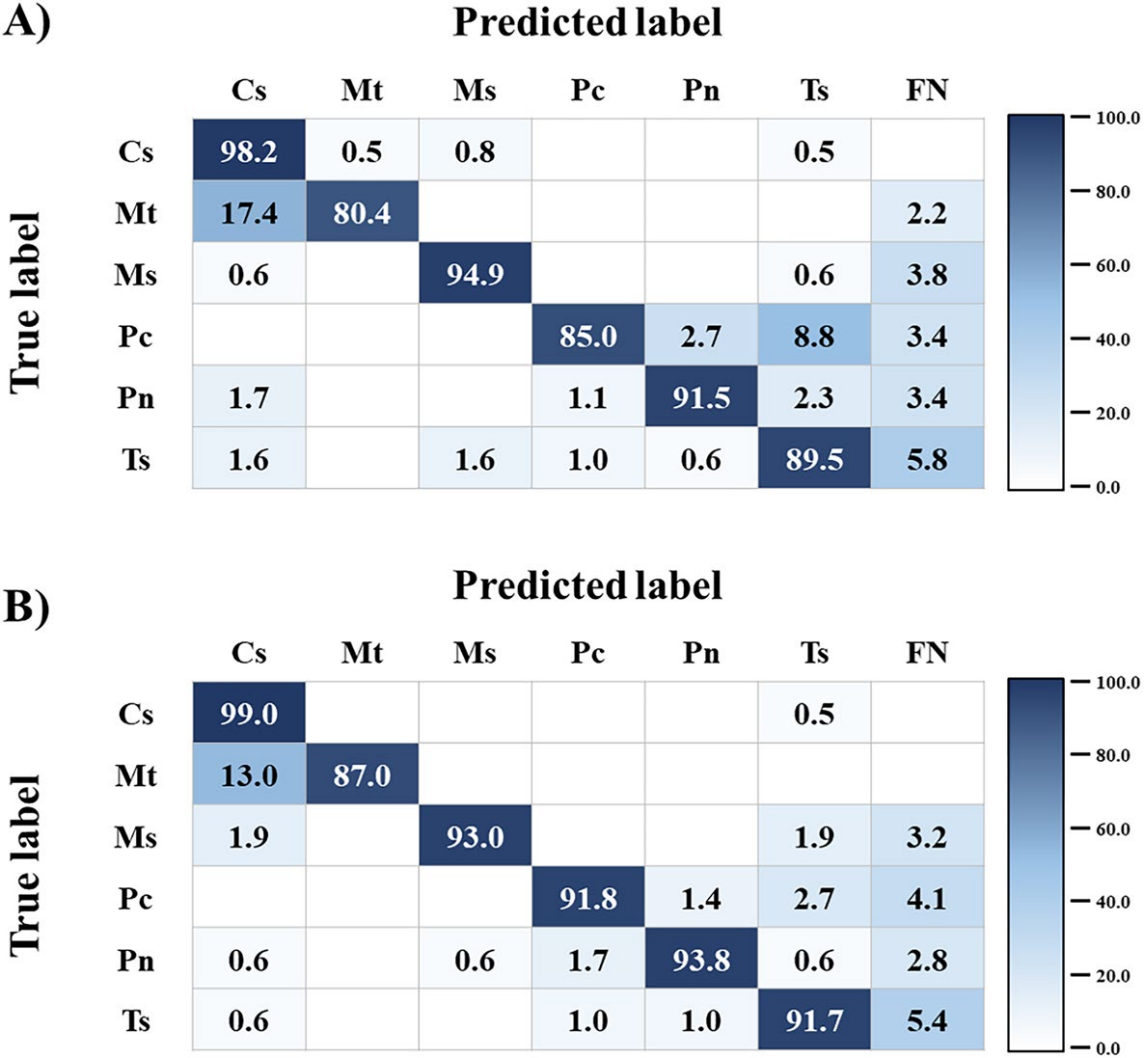
Confusion matrix illustrating the classification performance of the developed models for invasive turtle species. The classification rates are indicated by the color, with higher rates indicated by deeper shades of blue and lower rates appearing closer to white. **A**) Default model, **B**) Optimized model. Cs, *Chelydra serpentine*; Mt, *Macrochelys temminckii*; Ms, *Mauremys sinensis*; Pc, *Pseudemys concinna*; Pn, *Pseudemys nelsoni*; Ts, *Trachemys scripta*; FN, Background.

The classification results for six invasive turtle species, as determined using the two models, are presented as a confusion matrix in Fig. 5. The average correct classification rates across the six species was 89.9% for the default model, compared with 92.7% for the optimized model. For the default model, the correct classification rate was lowest for *M. temminckii* (80.4%) and highest for *C. serpentina* (98.2%; Fig. 5A). *M. temminckii* was most frequently misclassified as *C. serpentina*, with a misclassification rate of 17.4%. *P. concinna* was most commonly misclassified as *T. scripta* (8.8%), and *T. scripta* was mainly misclassified as background false negative (5.8%), which describes instances in which background regions were mistakenly identified as invasive turtle species, resulting in the false detection of non-existent objects. *C. serpentina*, which had the highest correct classification rate, was misclassified as *M. sinensis*, *M. temminckii*, and *T. scripta* in 0.8%, 0.5%, and 0.5% of images, respectively. In the optimized model, the correct classification rate ranged from 87.0% for *M. temminckii* to 99.0% for *C. serpentina* (Fig. 5B). In both models, the species with which *M. temminckii* and *T. scripta* were most frequently confused remained constant. However, the optimized model demonstrated a noticeable reduction in misclassification rates across several species. In particular, the rate of *M. temminckii* misclassification as *C. serpentina* decreased from 17.4% in the default model to 13.0% in the optimized model. Similarly, *T. scripta* was misclassified as background false negative in 5.8% of images in the default model, versus 5.4% following hyperparameter tuning. The most significant improvement was observed in the classification of *P. concinna*, for which the rate of misclassification as *T. scripta* dropped markedly from 8.8% to 2.7% in the optimized model. The largest improvement in correct classification was observed for *M. temminckii*, with the correct classification rate increasing from 80.4% to 87.0% after hyperparameter tuning. This improvement was largely attributable to the reduced rate of misclassification as *C. serpentina*.

## Discussion

Deep learning-based object detection models have displayed the ability to detect and classify species across various taxa^22,31^. Although these models have been successfully applied in this context, effective early detection of invasive species requires both robust object detection capabilities and high classification accuracy^49^. In this study, hyperparameter optimization with optimizer selection and hyperparameter tuning was applied to maximize mAP, a metric for evaluating both the detection and classification of invasive turtles. The results demonstrated that hyperparameter optimization, including optimizer selection and hyperparameter tuning, contributed to improved model performance. mAP0.5 reached 0.973, reflecting an improvement of 1.5% versus the default model (Table 4). Furthermore, mAP0.5–0.95, which provides a more comprehensive assessment across various levels of detection difficulty, increased by 3.2% to 0.841 (Table 4). Although direct comparison of mAP0.5–0.95 improvement with previous studies is limited by the lack of discussion of this metric in those works, the enhancement of mAP0.5 in this study represents a more substantial performance gain than in previous studies, where mAP0.5 either showed no meaningful improvement or increased by 0.9%^36,37^. These findings suggest that hyperparameter optimization is an effective strategy for improving model performance in the early monitoring of invasive species.

Four optimizers were evaluated: SGD, Adam, and the Adam variants Nadam and RAdam. SGD optimizer estimates the gradient of the loss function using small, randomly selected batches of data at each iteration^50^. Adam optimizer, which builds upon the RMSProp algorithm, combines adaptive learning rates and momentum and serves as the foundation for several other optimizers^51^. Although each optimizer offers specific advantages, SGD optimizer displayed the best overall performance in this analysis. Loss function analysis demonstrated that the SGD-applied model achieved the lowest values with the highest stability (Fig. 2) and the highest scores across all evaluation metrics (Fig. 3). This result suggests that the stochastic property of SGD was particularly well suited for detecting and classifying invasive turtles. This superiority might be attributable to the stochastic nature of the algorithm. SGD optimizer is known for its high generalization ability and its relatively fast escape from local minima^52^. In particular, high generalization performance might improve model performance and reduce the training time^53^. Although Adam optimizer has been widely used in biological detection and classification research^54,55^, SGD optimizer yielded superior performance for the invasive turtle dataset in this study. This performance difference might be attributable to the tendency of the Adam-applied model to exhibit appropriate performance during relatively short training^56^. The SGD-applied model demonstrated the best performance at epoch 258 among the 300 epochs conducted in this study. Therefore, selecting the most suitable optimizer for the target dataset could play a significant role in optimizing model performance. Furthermore, the observed performance gap between the best- and worst-performing models highlights the importance of optimizer selection as a critical first step in hyperparameter optimization.

In the hyperparameter tuning process, 16 hyperparameters were adjusted in the SGD model using random search. Among the 16 hyperparameters, five varied by more than 30%, including three training-related parameters (cls, weight_decay, and lr0) and two augmentation-related parameters (mosaic and hsv_v). Regarding the training-related parameters, cls exhibited the most significant adjustment relative to its default value (Table 3). This parameter determines the weight assigned to the classification loss function, and its upward adjustment indicates that greater emphasis was placed on accurate class prediction^57^. weight_decay, which controls the strength of L2 regularization, also increased after hyperparameter tuning. This adjustment implies that stronger regularization was applied to reduce the risk of overfitting^57^. Meanwhile, lr0 decreased after optimization. This reduction was intended to slow convergence slightly in favor of more stable training dynamics^57^. The three training-related parameters were tuned to improve training stability and classification accuracy. Indeed, this optimized model achieved stable learning without overfitting (Fig. 4), with improvements in classification accuracy visible in the confusion matrix (Fig. 5). This suggests that hyperparameter tuning of training-related hyperparameters might actually improve model robustness and classification performance. Concerning augmentation parameters, the mosaic parameter defines the probability of combining four images into a single synthetic sample. A reduction in this probability suggests that overly complex composite images could diverge from the true data distribution or introduce unwanted noise^58^. hsv_v controls the range of brightness variation during augmentation. A narrower range indicates more restrained augmentation, helping to preserve the original luminance characteristics of the images^57^. The two augmentation-related parameters were constrained to ensure that the augmented images remained representative of the original data, thereby minimizing the risk of distortion during learning. As listed in Table 4, the application of these adjusted values led to an improvement in mAP, indicating enhanced model performance. These results underscore the importance of the original color information in training models for invasive turtles and suggest that limiting excessive augmentation techniques could lead to more effective learning.

In the confusion matrix analysis, the correct classification rate for *P. concinna* was 91.8% in the optimized model, versus 85.0% in the default model (Fig. 5). This represented the most substantial improvement in classification accuracy among the six invasive turtle species following hyperparameter optimization. The improvement was primarily attributable to a reduction in the misclassification of *P. concinna* as *T. scripta* after hyperparameter tuning. Although these two species should be distinguished by the presence or absence of two reddish or orange bands behind the eyes and above the ear flaps^40,42^, the default model misclassified *P. concinna* as *T. scripta* in 8.8% of images. However, this misclassification was reduced through the application of hyperparameter settings that were optimized to the dataset developed in this study. Moreover, the optimized model reduced the misclassification of *M. temminckii* as *C. serpentina* relative to the default model (Fig. 5). Although these two species might be distinguished by the presence or absence of a scute between the costal and marginal scutes on both sides, these species remain challenging to accurately distinguish because of their similar morphological characteristics, particularly the presence of three keels on the carapace^40–42^. Despite the observed reduction in misclassification, the misclassification rate between these two species remained higher than that between any other species pair in both models. Morphological similarity was previously identified as the primary factor contributing to species misclassification by deep learning models^59–61^. Furthermore, this persistent misclassification could stem from the relatively smaller number of *M. temminckii* images in the training set relative to the other species^61^. Therefore, to enhance classification accuracy for *M. temminckii*, additional training images clearly depicting the region between the costal and marginal scutes on both sides of the carapace should be incorporated into the dataset.

Overall, the optimized model outperformed the default model in terms of mAP for detecting and classifying invasive turtles. This result suggest that hyperparameter optimization might permit model performance to be enhanced without additional data. This could represent an effective strategy for improving performance in cases in which further data collection is particularly difficult, such as the case for rare or endangered species. Furthermore, this method can be easily combined with other strategies designed to improve model performance, such as transfer learning, architectural enhancements, and augmentation methods that improve image clarity^62–64^. Using these complementary methodologies alongside hyperparameter optimization could maximize model performance more effectively than using a single method. Despite these performance improvements and the utility of the approach, improvements to further enhance model performance for classifying invasive turtles would be beneficial for monitoring these species. In this study, the classification accuracy for *M. temminckii* remained relatively lower than that for other species. This reduced accuracy could stem from an insufficient and unbalanced dataset^60,61^. Recently, generative adversarial network (GAN) models have been effectively utilized to generate synthetic image data for wildlife, including rare species^65,66^. These models have proven useful for expanding and balancing datasets, thereby enhancing the performance of wildlife classification models. Therefore, future studies should apply GAN models to augment the dataset for *M. temminckii*, potentially improving its classification performance.

Moreover, deep learning models continue to advance rapidly, as exemplified by the recent development of YOLOv12^67^. This model integrates area attention, the residual efficient layer aggregation networks (R-ELAN), and architectural optimizations to achieve better accuracy and efficiency^67^. Therefore, future research should apply such cutting-edge models to classify invasive turtles to further enhance model performance and improve classification accuracy. Furthermore, accurate and efficient counting of individuals is critical for the early detection and management for invasive species^68^. Expanding the model to include object counting functionality might enable real-time monitoring of invasive turtles by a few field researchers. The final model might be loaded into camera traps and drones through future studies assessing hardware feasibility, thereby enabling early detection across broad survey areas.

## Conclusion

This study applied hyperparameter optimization, including optimizer selection and hyperparameter tuning, to improve model performance in detecting and classifying six invasive turtle species in Korea. The optimized model outperformed the default model. Specifically, AP was improved for all six species in the optimized model, accompanied by a reduction in misclassification rates and an overall enhancement in classification accuracy. The optimized model holds promise for supporting the early detection and management of invasive turtles. Furthermore, continued advancements in deep learning-based detection and classification models for invasive turtles represent an important step toward the conservation of biodiversity.

## Supplementary Information

Below is the link to the electronic supplementary material.

Supplementary Material 1

## Data Availability

All data except images the authors do not have the right to share and trained models are available from the Kaggle repository (https://www.kaggle.com/datasets/bjh03205/invasive-turtle-dataset).
