# Supplementary material for: Hyperparameter optimization to enhance the performance of deep learning models for the early detection of invasive turtles in Korea
Source: Sci Rep. 2026 Feb 6;16:7561. doi: 10.1038/s41598-026-37636-2 (PMC12932630; doi:10.1038/s41598-026-37636-2)
Supplement: Supplementary file 1 — Supplementary Material 1 [file 41598_2026_37636_MOESM1_ESM.docx]

**Supplementary Materials**

**Hyperparameter optimization to enhance the performance of deep learning models for the early detection of invasive turtles in Korea**

Jong-Won Baek^1^†, Jung-Il Kim^2^†, Min-Ho Mun^1^ and Chang-Bae Kim^1*^

^1^Biotechnology Major, Sangmyung University, Seoul 03016, Korea

^2^Ocean Climate Response and Ecosystem Research Department, Korea Institute of Ocean Science and Technology, Busan 49111, Korea

†These authors contributed equally to this work.

*****Email: evodevo@smu.ac.kr

**Table S1.** Brief description of the six optimizers.

| **Optimizer** | **Description** |
| --- | --- |
| Stochastic Gradient  Descent (SGD) | Updates model parameters incrementally in the direction of the negative gradient of the loss function, proportionate to a learning rate |
| Adaptive moment estimation (Adam) | An updated model's weights that combines momentum with per-parameter learning rate adjustment, using both gradient magnitudes and past gradient information |
| Adam with decoupled weight decay (AdamW) | A modified version of Adam that included weight decay from the loss function |
| Nesterov-accelerated  Adam (Nadam) | An extension of Adam that integrates Nesterov-accelerated gradient momentum |
| Rectified Adam (RAdam) | A variant of Adam that introduces gradients variance-based adaptive learning rate rectification |
| Root Mean Square Propagation (RMSProp) | A momentum-based optimizer that adapts individual learning rates by utilizing a moving average of squared gradients |

**Table S2.** Predefined ranges of 16 hyperparameters used in this study.

| **Hyperparameter** | **Predefined ranges** | |
| --- | --- | --- |
|  | **Minimum** | **Maximum** |
| lr0 | 0.00001 | 0.1 |
| lrf | 0.01 | 1.0 |
| momentum | 0.6 | 0.98 |
| weight_decay | 0.0 | 0.001 |
| warmup_epochs | 0.0 | 5.0 |
| warmup_momentum | 0.0 | 0.95 |
| box | 0.02 | 0.2 |
| cls | 0.2 | 4.0 |
| dfl | 0.4 | 6.0 |
| hsv_h | 0.0 | 0.1 |
| hsv_s | 0.0 | 0.9 |
| hsv_v | 0.0 | 0.9 |
| translate | 0.0 | 0.9 |
| scale | 0.0 | 0.9 |
| fliplr | 0.0 | 1.0 |
| mosaic | 0.0 | 1.0 |

**Table S3.** Comparison of loss function results at the best-performing epoch for the examined models.

| **Metrics** | **Default model** | **Optimized model** |
| --- | --- | --- |
| Complete Intersection over Union loss (*LCIoU*) | 0.61014 | 0.49867 |
| Classification loss (*Lcls*) | 0.37940 | 0.50189 |
| Distribution focal loss (*Ldfl*) | 1.09574 | 0.96152 |


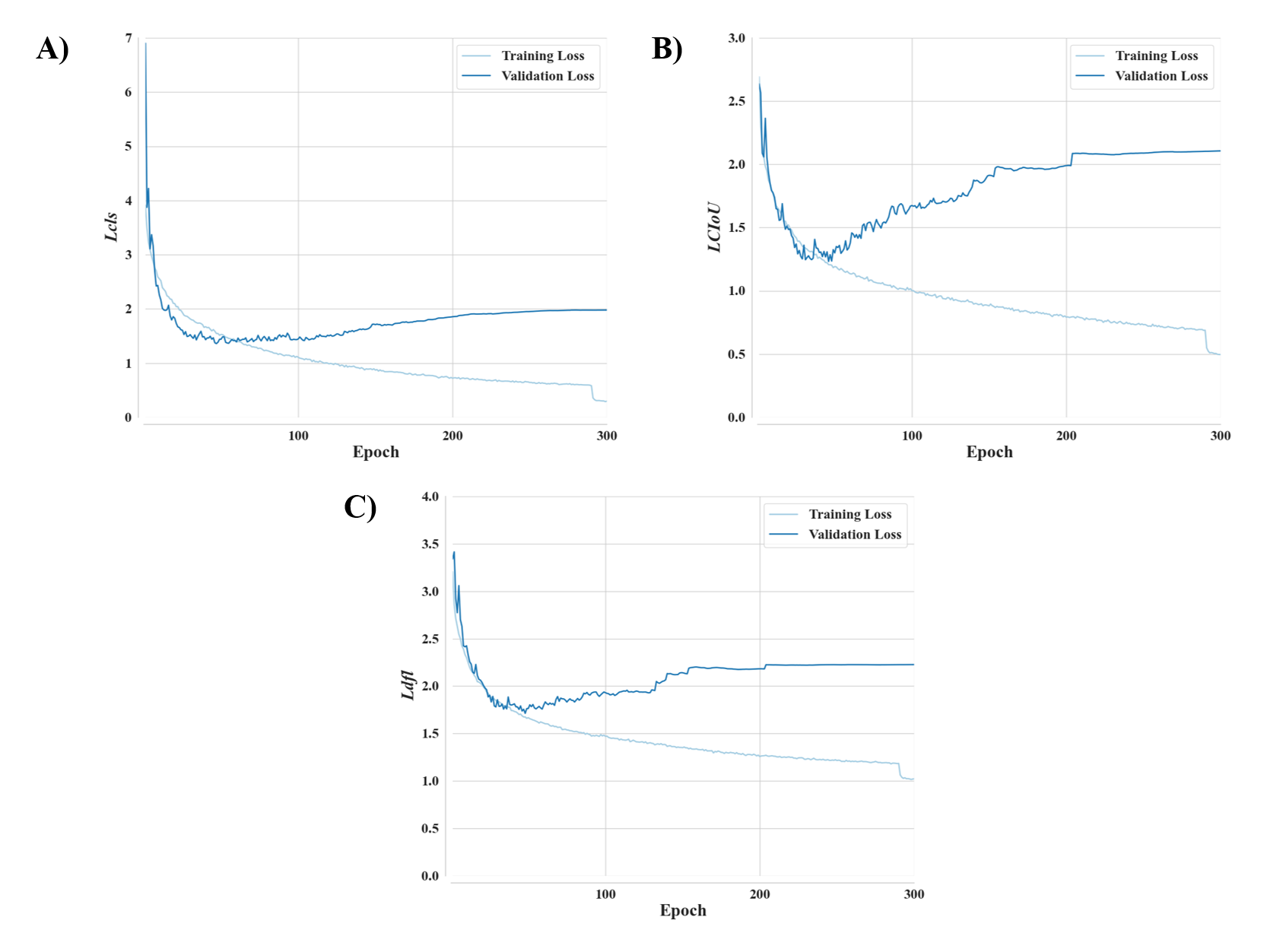


**Figure S1.** Loss function results of AdamW model. A) Complete Intersection over Union loss (*LCIoU*), B) Classification loss (*Lcls*), C) Distribution focal loss (*Ldfl*).


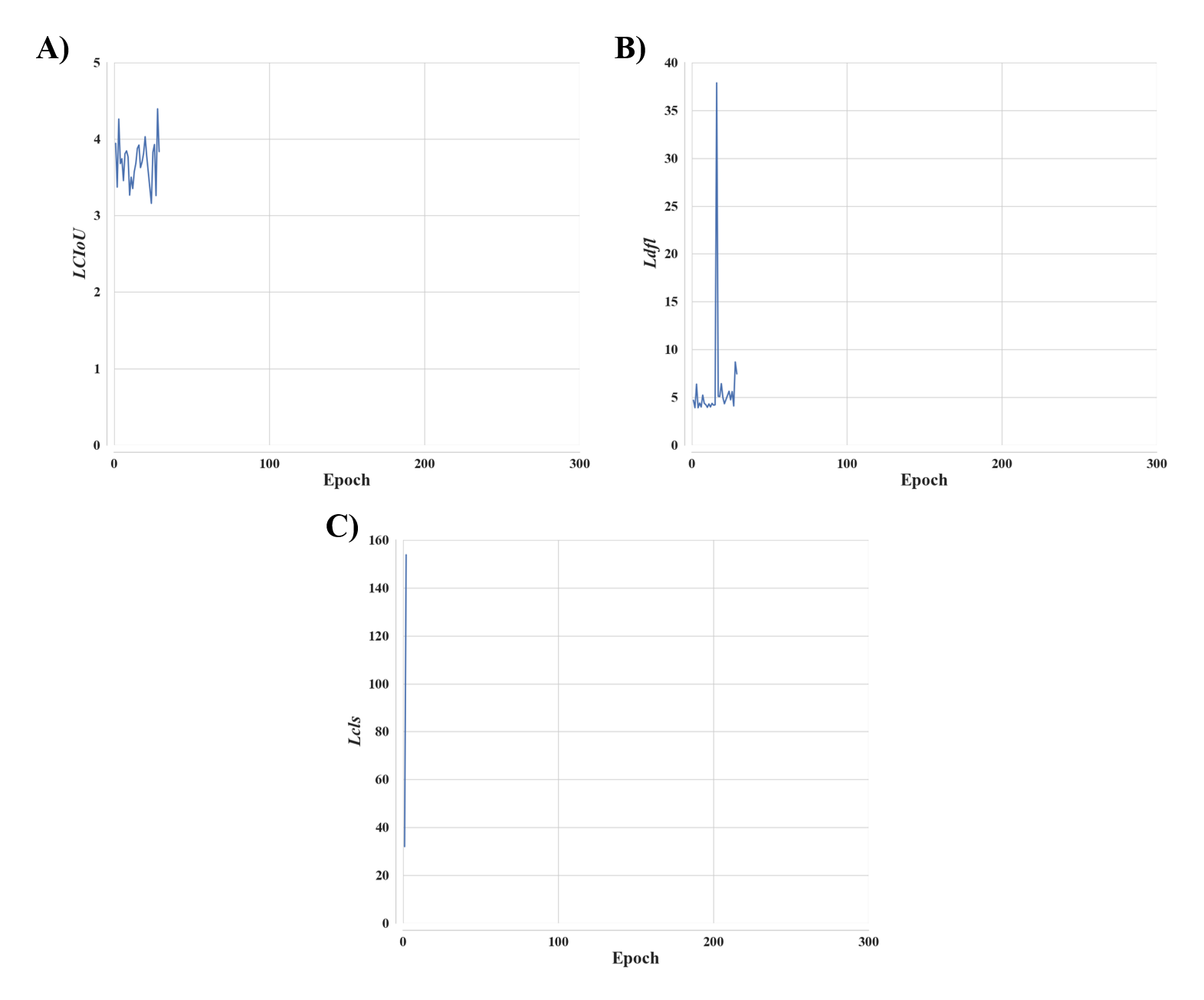


**Figure S2.** Loss function results of the RMSProp model. A) Complete Intersection over Union loss (*LCIoU*), B) Classification loss (*Lcls*), C) Distribution focal loss (*Ldfl*).

**
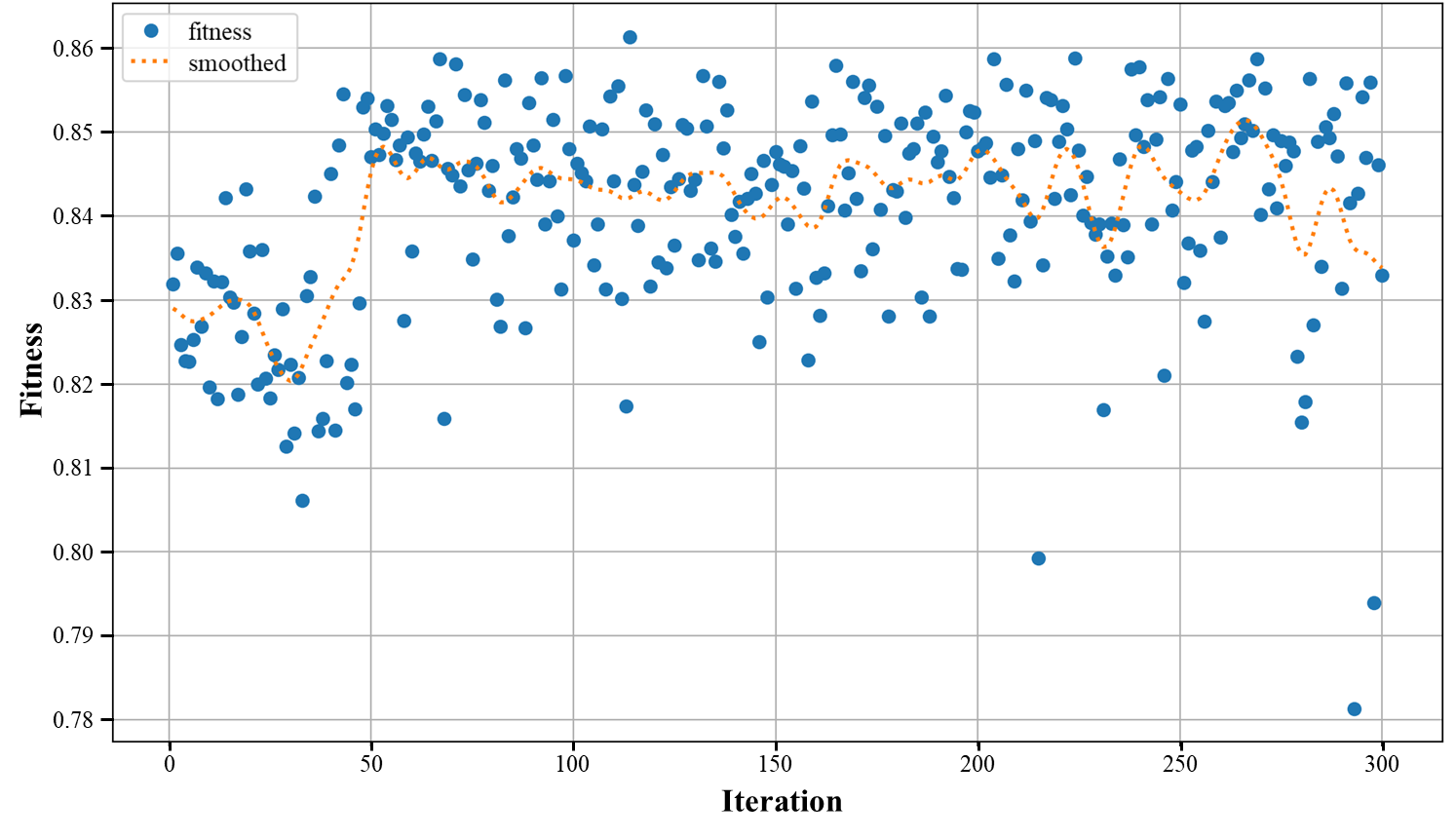
**

**Figure S3.** Fitness scores plotted as a function of the number of iterations hyperparameter tuning.

*
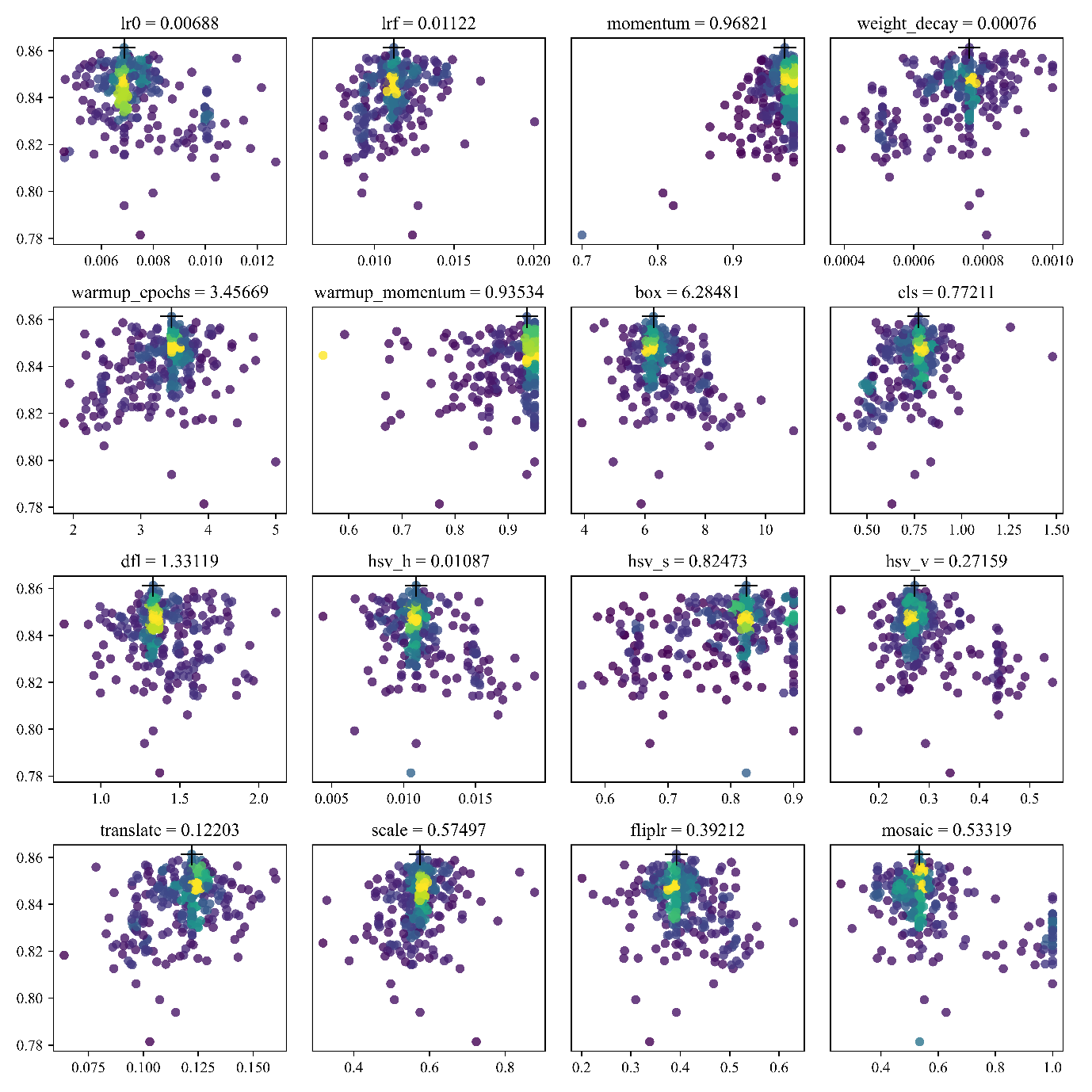
*

**Figure S4.** Scatter plots presenting hyperparameter convergence during hyperparameter tuning.
